# Methylglyoxal Formation—Metabolic Routes and Consequences

**DOI:** 10.3390/antiox14020212

**Published:** 2025-02-13

**Authors:** Janka Vašková, Gabriela Kováčová, Jakub Pudelský, Drahomír Palenčár, Helena Mičková

**Affiliations:** 1 Department of Medical Biology, Faculty of Medicine, Pavol Jozef Šafárik University, 040 11 Košice, Slovakia; 2 Department of Medical and Clinical Biochemistry, Faculty of Medicine, Pavol Jozef Šafárik, 040 11 Košice, Slovakia; gabriela.kovacova@upjs.sk (G.K.);; 3 Department of Plastic Surgery, Faculty of Medicine, Comenius University Bratislava, 813 72 Bratislava, Slovakia

**Keywords:** advanced glycation end products, glutathione, lipoxidation, methylglyoxal, reactive carbonyl species, reactive oxygen species

## Abstract

Methylglyoxal (MGO), a by-product of glycolysis, plays a significant role in cellular metabolism, particularly under stress conditions. However, MGO is a potent glycotoxin, and its accumulation has been linked to the development of several pathological conditions due to oxidative stress, including diabetes mellitus and neurodegenerative diseases. This paper focuses on the biochemical mechanisms by which MGO contributes to oxidative stress, particularly through the formation of advanced glycation end products (AGEs), its interactions with antioxidant systems, and its involvement in chronic diseases like diabetes, neurodegeneration, and cardiovascular disorders. MGO exerts its effects through multiple signaling pathways, including NF-κB, MAPK, and Nrf2, which induce oxidative stress. Additionally, MGO triggers apoptosis primarily via intrinsic and extrinsic pathways, while endoplasmic reticulum (ER) stress is mediated through PERK-eIF2α and IRE1-JNK signaling. Moreover, the activation of inflammatory pathways, particularly through RAGE and NF-κB, plays a crucial role in the pathogenesis of these conditions. This study points out the connection between oxidative and carbonyl stress due to increased MGO formation, and it should be an incentive to search for a marker that could have prognostic significance or could be a targeted therapeutic intervention in various diseases.

## 1. Introduction

**Figure 1:**
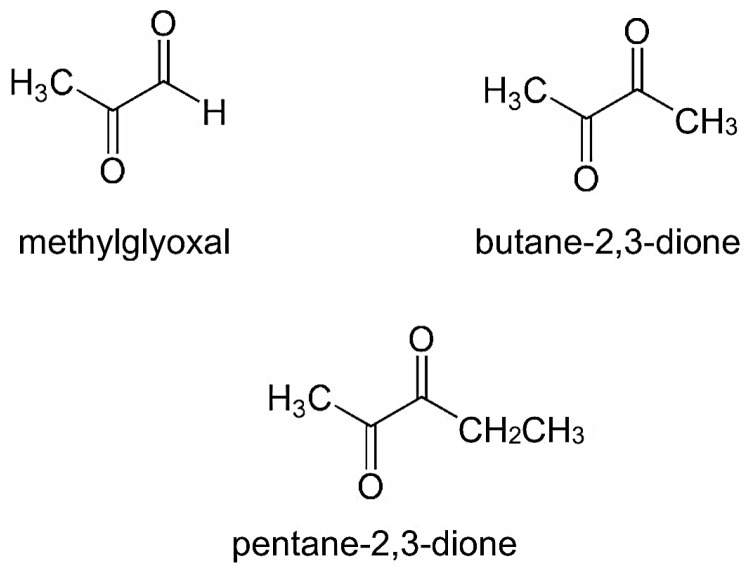
A-dicarbonyl compounds. The formation of methylglyoxal is part of the metabolic processes in humans and mammals in general. The occurrence of the other two dicarbonyl compounds in the body is determined by the intake from dietary sources, where they are often not only a product of the bacterial fermentation of saccharides but also an indicator of the oxidation or bacterial contamination of food.

Methylglyoxal (MGO), also known as 2-oxopropanal or pyruvaldehyde, is an electrophilic dicarbonyl compound that is commonly formed as a by-product of cellular metabolism. It is produced under both physiological and pathological conditions [1], and it is formed during the oxidation of hexoses by the conversion of triose phosphates, which reduces the formation of ATP. At the intracellular level, it is formed in an amount of approximately 1–4 μM [2], not only deriving from glycolysis but also from the metabolism of ketone bodies, lipids, and the amino acids glycine and threonine (also the decarboxylation of alanine and hydroxylation of taurine) from acetone, hydroxyacetone (acetol), and aminoacetone, respectively. Enzymatically, the conversion is catalyzed by cytochrome P450 (CYP2E1), acetol monooxygenase, myeloperoxidase, and the semicarbazide-sensitive amine oxidase [3]. In a 70 kg man, with a daily MGO production of 3 mg per kg of body weight [4], approximately 0.089% of the MGO is derived from triose phosphates (in erythrocytes). By contrast, up to 3% is produced through the conversion of threonine [5], and 7% originates from glycosylated proteins and the degradation of monosaccharides [6]. During prolonged fasting, low-calorie diets, or diabetes, ketone bodies become a significant source of MGO [7]. Consequently, the formation of MGO, as well as other α-dicarbonyl compounds, can be influenced by dietary sources (Figure 1) [8].

This paper discusses the mechanisms organisms use to maintain the levels of MGO, as well as the consequences of the predominance of the formation of reactive compounds from MGO in animal model organisms and in humans. A literature search was conducted across several academic databases, including PubMed, Scopus, and Web of Science, to ensure an overview of the relevant research. This was performed using a combination of specific keywords related to the topic, including oxidative stress, reactive oxygen species (ROS), carbonyl stress, methylglyoxal, glyoxalase 1 and 2, glutathione, antioxidants, advanced glycation end products (AGEs), signaling pathways, and disease pathology. Boolean operators were applied to refine the search strategy and ensure the inclusion of relevant studies. The time frame for conducting the literature search was from 2023 to 2024, focusing on peer-reviewed articles, reviews, and experimental studies. The keywords were selected to cover a broad range of topics, from general oxidative stress mechanisms to specific molecular pathways, such as the glyoxalase system and its role in detoxifying methylglyoxal, as well as the formation of AGEs and their impact on disease development.

## 2. Meaning and Implications of Methylglyoxal Formation

**Figure 2:**
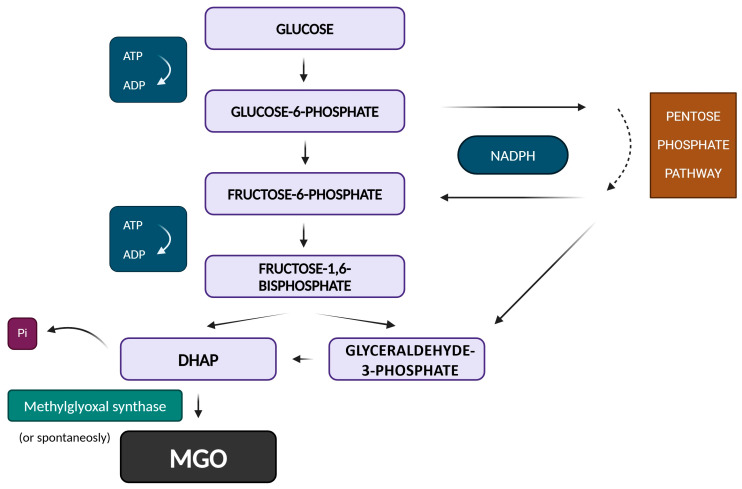
The pathway for the formation of methylglyoxal (MGO) from triose phosphates in anaerobic glycolysis.

However, what makes the formation of MGO inevitable is the properties of the intermediate product of glycolysis itself—dihydroxyacetone phosphate (DHAP). It follows from the process of MGO synthesis that in anaerobic glycolysis, it represents a diversion from ATP synthesis and, thus, energy generation does not occur, while preserving the possibility of a hexose monophosphate shunt for NADPH formation (Figure 2). Even DHAP represents a branch for the synthesis of triacylglycerols using NADPH instead of ATP formation. An important characteristic of DHAP is its relatively low chemical stability. Degradation begins with deprotonation. However, instead of re-protonation to form glyceraldehyde 3-phosphate, the phosphate group is released. This degradation reaction also occurs as a side reaction during triose phosphate isomerase catalysis [9]. The typical chemical half-lives of DHAP range from about 3 h at 37 °C to 30 h at 25 °C under neutral to slightly basic conditions [10]. MGO was thought to be formed non-enzymatically; however, in the 1970s, during studies of several bacterial organisms, it was found that the formation of MGO is catalyzed by methylglyoxal synthase (MGS) with a *K*_m_ of 0.47 mM and an optimum pH of 7.5 [11,12]. MGS (E.C. 4.2.3.3) removes phosphate, forming pyruvaldehyde (MGO) [13].

Finally, MGO can be metabolized to D-lactate and pyruvate. Similarly to the pentose phosphate pathway, which leads to the synthesis of NADPH and precursors for the synthesis of nucleotides, and DHAP, which leads to the synthesis of triacylglycerols, the formation of MGO is also expected as a necessary metabolic diversion in the case of an excess of saccharides. In addition, it serves as a source of phosphate for the cell and can also function as a phosphate sensor [1]. The methylglyoxal pathway appears to have its justification in an excess of energy-generating sources.

On the other hand, in vivo, millimolar concentrations of MGO were found to be mutagenic and to disrupt the de novo synthesis of proteins and nucleic acids [14] through glycation and to increase the level of dicarbonyl stress. The increased level of stress conditions with higher concentrations of MGO is also supported by the release of Pi from triosephosphate. In certain human tissues, including the brain, liver, and heart, Pi has been shown to trigger the release of mitochondrial ROS. As the primary anion permeable to intracellular membranes, Pi can disrupt the mitochondrial pH gradients (∆pH) via the mitochondrial H+/Pi cotransporter. This, in turn, increases the membrane potential (∆Ψm) and promotes ROS production [15].

Maintaining low levels of MGO by its metabolism through glutathione-dependent and -independent pathways is crucial, particularly in tissues with high energy demands, as dicarbonyl stress—resulting from increased MGO formation—plays a significant role in the pathophysiology of various diseases.

### 2.1. Return to the End Product of Anaerobic Glycolysis

Methylglyoxal (MGO) is a potent glycotoxin and a key precursor of advanced glycation end products (AGEs) [16], which are linked to various pathological conditions, including aging and neurodegenerative diseases [1]. Under physiological conditions, cells effectively eliminate MGO through various mechanisms, with the glyoxalase system being the primary pathway for its detoxification [17] and being present in all cells [18].

MGO non-enzymatically reacts with glutathione (GSH) to form a hemithioacetal, which is subsequently recognized by glyoxalase 1 (GLO1, lactoylglutathione lyase; EC 4.4.1.5). GLO1 then converts the hemithioacetal into S-D-lactoylglutathione, which is later recognized by glyoxalase 2 (GLO2, EC 3.2.1.6) and hydrolyzed to d-lactate [19]. An alkaline pH of around 8 is generally the most optimal for GLO1. GLO1 has the ability to convert various glutathione hemithioacetals to glutathione thioesters [20]. Its activity can be inhibited by acylation and modulated by phosphorylation or nitrosylation [21]. Other compounds that influence MGO activity include 4-(7-azaindole)-substituted 6-phenyl-N-hydroxypyridones, flavonoids, S-bromobenzylglutathione cyclopentyldiester, and curcumin [22,23], and it can also be affected by ionizing radiation [24] and an NO [25]. GLO2 (hydroxyacylglutathione hydrolase) is a thioesterase catalyzing the hydrolysis of glutathione substrates to d-lactate and GSH under alkaline conditions [26,27]. GLO3 converts methylglyoxal (MG) into d-lactate in a single step, without the involvement of GSH [28]. GLO3 contains DJ-1_PfpI domains that allow the enzyme to act as a protease, chaperone, deglycase, and esterase, and it performs other physiological functions in transcription, mitochondrial regulation [29,30], and oxidative stress signalization [31].

The glutathione pathway has its pros and cons. GLO1 activity can be considered detoxifying as it forms a non-toxic product, S-D-lactoylglutathione, but is not indispensable, given the MGO elimination pathways described below. Kant et al. [32] confirmed the equal importance of GLO2 in promoting *Salmonella*’s resistance to oxidative stress. There is a literal switching of glycolysis to the formation of MGO in order to maintain the glucose intake and aerobic oxidation. GSH then neither undergoes a spontaneous reaction with the hydrogen peroxide formed nor is depleted in reactions with the products of the host cell phagocyte NADPH oxidase. The oxidized form requiring a system of reductases is not formed. It is bound to S-D-lactoylglutathione, from where it is released via GLO2. By contrast, the overexpression of GLO1 is associated with growth, the progression of several types of cancer, and resistance to therapy [33,34,35], as bypassing glycolysis allows tumor cells to promote increased demands for NADPH in maintaining the redox state.

**Figure 3:**
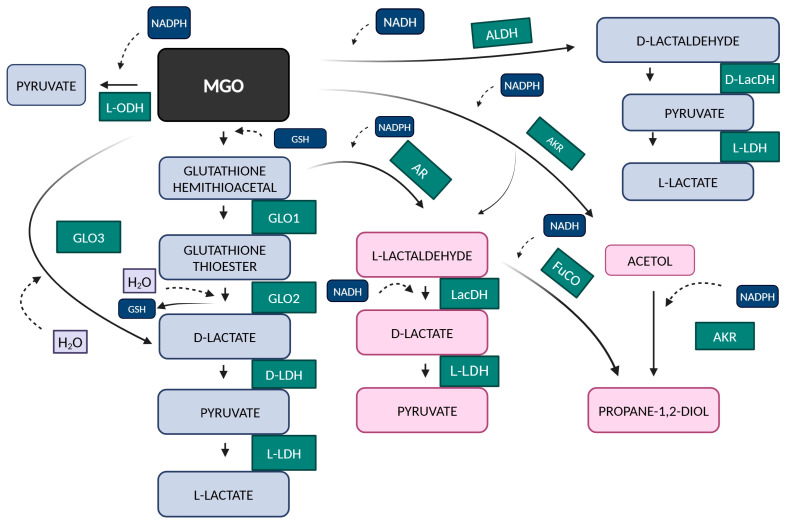
The biochemical processes involved in the metabolism of methylglyoxal (MGO). In the glutathione pathway, the hemithioacetal formed non-enzymatically by glyoxalase 1 (GLO1) is converted into S-D-lactoylglutathione (a thioester). This compound is then processed by glyoxalase 2 (GLO2), which releases glutathione (GSH) and forms D-lactate, which can be further converted by lactate dehydrogenase (LDH). In addition, the non-glutathione pathways also convert methylglyoxal (MGO) through enzymes such as glyoxalase 3 (GLO3). By an oxidative pathway via 2-oxoaldehyde dehydrogenase (2-ODH) or aldehyde dehydrogenase (ALDH), conversion by reduction is also possible via aldo-keto reductases (AKRs), aldose reductase (AR), and the catalytic activity of lactaldehyde dehydrogenase (LacDH) and 1,2-propanediol oxidoreductase (FuCO).

Other ways of MGO detoxification independent of glutathione according to [12,36] are summarized in Figure 3. MGO is then, through oxidation and reduction processes in the course of dehydrogenases, transformed into carboxylic acids and into lactaldehyde and acetol by aldo-keto reductases [2]. AKRs exhibit distinct kinetic and expression profiles in response to varying intracellular glyoxal concentrations. In humans, the aldose reductase family (ALR) consists of three enzymes that catalyze the conversion of various carbonyl substrates. Of these, ALR2 exhibits the highest specificity for methylglyoxal. At physiological concentrations of GSH, approximately 3 mM, the activities of GLO1 and ALR2 converge, targeting methylglyoxal/hemithioacetal substrates. Notably, ALR2-mediated detoxification becomes predominant at lower GSH concentrations [37].

AKR gene polymorphisms are associated with the development of retinopathy, nephropathy, and neuropathy in diabetic patients [38,39]. The overexpression of AKR genes or proteins contributes to the drug resistance observed in cancers, inflammatory conditions, and neural atrophy [40,41,42]. Mutations in ALDH genes are also linked to various conditions, including heart disease, muscular dystrophy, and neuroinflammation [43,44,45].

### 2.2. Lactate or Propane-1,2-Diol

Both pathways of MGO metabolism lead to the formation of several distinct final products. The accumulation of L-lactate in the human body is affected by metabolic processes and can lead to lactic acidosis [46]. Therefore, lactate must be rapidly cleared from tissues and circulation to prevent harm. The irreversible removal of lactate is primarily facilitated by pyruvate dehydrogenase. Additionally, lactate accumulation can stimulate gluconeogenesis in the liver and skeletal muscle cells, where lactate is converted into glucose and released into the bloodstream to support further glucose consumption during periods of increased energy expenditure [47,48]. However, lactate has also been identified as a signaling molecule, exerting its effects through the G-protein-coupled receptor 81 (GPR81). Additionally, lactate is transported into cells via monocarboxylate transporters (MCTs), which facilitate its cellular uptake [49,50]. Lactate accumulation in the tissue is a hallmark of inflammatory diseases and tumor cells. However, mounting evidence indicates that the Warburg effect, where cells preferentially ferment glucose to lactate rather than fully oxidizing it to CO_2_, also occurs in many non-tumor cells. This metabolic shift has been observed in a variety of non-cancerous diseases [51,52]. It is likely that lactylation, as an epigenetic modification, also plays a significant role in these conditions [53].

d-lactaldehyde can be converted into d-lactic acid through an alternative metabolic pathway in the liver, as it does not serve as an effective substrate for aldehyde dehydrogenase. This pathway involves reduced glutathione as a cofactor. Unlike L-lactic acid, which is metabolized more rapidly, d-lactic acid tends to accumulate in the body due to its slower rate of metabolism [54]. Under normal conditions, it is produced in minimal amounts in human tissues and is undetectable in the bloodstream [55]. d-lactate is seldom found in the brain and does not serve as an energy source [56]. The rate of D-lactate oxidation in the brain is significantly lower than that of L-lactate, primarily due to the low expression of d-lactate dehydrogenase (d-LDH) in brain tissue [57]. d-lactate metabolism primarily occurs in the mitochondria through the action of d-lactate dehydrogenase. This FAD/FMN-dependent enzyme, located on the inner surface of the inner mitochondrial membrane, oxidizes d-lactate to pyruvate in the mitochondrial matrix. It also transfers reducing equivalents by oxidizing d-lactate to coenzyme Q, which then passes them to complex III of the respiratory chain [58,59]. d-lactate is excreted from cells [60,61] or even recycled back to MGO [62]. Certain probiotics, such as *Lactobacillus* species, exhibit d-LDH activity, which may be beneficial in treating d-lactic acidosis [63]. The physiological role of d-lactate is now understood as that of a competitive inhibitor of L-lactate, disrupting the utilization of more efficient energy substrates like pyruvate and L-lactate, potentially negatively impacting energy metabolism [64].

**Figure 4:**
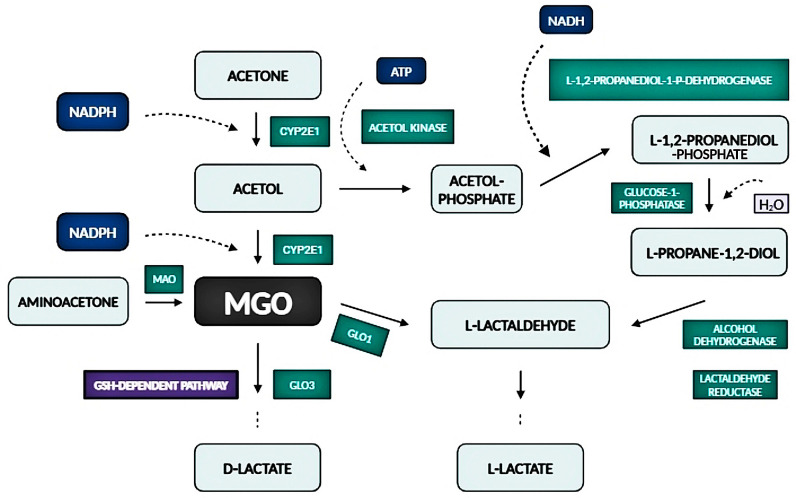
The formation and detoxification of methylglyoxal (MGO) from acetone and aminoacetone.

Another metabolite is propane-1,2-diol. As can be seen from Figure 3, methylglyoxal is converted to acetol through the NADPH-dependent methylglyoxal reductase belonging to the aldo-keto reductase (AKR) superfamily. As shown in other studies, this product mostly arises from acetone [65] but is not always a product of MGO metabolism. The metabolic peculiarities of acetone are significant as higher concentrations are produced during starvation or in cases of diabetes mellitus. The initial process of the conversion of acetone to acetol by a cytochrome P450 isoenzyme (Figure 4) is induced by acetone itself [66]. Cytochrome P450 isoenzymes exhibit tissue-specific and sex-dependent differences in expression [67]. In addition, the metabolism of acetone is further diversified depending on its concentration. Concentrations of acetone lower than 4 mM predominate in the pyruvate pathway, and concentrations above this favor MGO formation. The addition of acetone resulted in the detection of acetol and propane-1,2-diol in the plasma of male rats; however, only propane-1,2-diol was detected when acetol was administered [68]. It was revealed that glucose was synthesized in the hepatocytes of male rats exposed to increased acetone levels by administering 1% acetone in drinking water for 5–6 days, followed by 48 h of fasting. Additionally, the inclusion of acetol in the hepatocyte incubation medium also induced the synthesis of glucose and D-lactate. The metabolites MGO and propane-1,2-diol were further utilized for glucose synthesis [69]. Glucose synthesis from acetone at high concentrations (20 mM) was not confirmed in murine hepatocytes from pretreated rats subjected to 48 h of starvation [70], nor was glucose synthesis observed from acetol. Similarly, gluconeogenesis from both acetone and acetol was not confirmed in streptozotocin-induced diabetic male mice [71]. However, in both cases, glucose formation was measured from MGO [70,71]. The concentrations of methylglyoxal and D-lactate have not been measured in humans during fasting [65]. An explanation for this could be found in Salomón et al. [72]. The reaction of MGO with acetoacetate leads to the formation of 3-hydroxyhexane-2,5-dione. This compound primarily undergoes reductive metabolism in human blood, resulting in the production of non-glycating species. It is also interesting that in diabetic patients, the conversion of acetone to acetol and propane-1,2-diol was confirmed [73].

## 3. Carbonyl and Oxidative Stress

Carbonyl stress arises when there is an imbalance between the production and the metabolism of dicarbonyl metabolites, including those derived from external sources [74]. Reactive carbonyl species (RCS) are also formed when monosaccharides react with oxygen radicals. Dicarbonyl saccharide 3-deoxyglucosone results in glucose oxidation by a hydroxyl radical [75]. It can be inferred that, similar to the previously discussed pathways for MGO formation, this represents a comparable radical transformation, leading to the production of α-oxoaldehydes [76]. A-dicarbonyls (as well as MGO) are highly chemically reactive—up to 20,000 times more reactive than glucose—with the amino groups of the biomolecule [77,78]. Glycation mediated by α-dicarbonyl compounds occurs several orders of magnitude faster than glycation by glucose [79,80]. The glycation of biopolymers by reactive carbonyl species, a process known as the Maillard reaction, results in the formation of advanced glycation end products (AGEs) [81].

**Figure 5:**
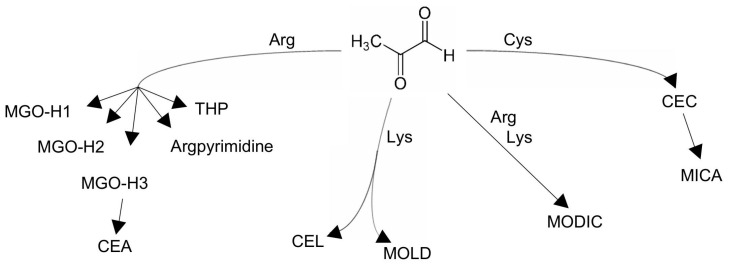
Methylglyoxal in reaction with arginine residues forms methylglyoxal-hydroimidazolones (MGO-H1, MGO-H2, MGO-H3, or N_ɛ_-(5-hydro-5-methyl-4-imidazolon-2-yl)-l-ornithine; 2-amino-5-(2-amino-5-hydro-5-methyl-4-imidazolon-1-yl) pentanoic acid; 2-amino-5-(2-amino-4-hydro-4-methyl-5-imidazolon-1-yl) pentanoic acid), argpyrimidine (N_δ_-(5-hydroxy-4,6-dimethylpyrimidine-2-yl)-l-ornighine), and THP (*N*_δ_-(4-carboxy-4,6-dimethyl-5,6-dihydroxy-1,4,5,6-tetrahydropyrimidine-2-yl)-L-ornithine). MGO-H3 is further able to form CEA (*N*^ω^-carboxyethyl-arginine) by hydrolysis. The reaction of MGO with lysine residues results in CEL (Nɛ-(carboxymethyl)-l-lysine) and MOLD (methylglyoxal lysine dimer) formation. MODIC (methylglyoxal-derived imidazolium crosslink derived from methylglyoxal and lysine-arginine) arises in the reaction of arginine and lysine residues with MGO. MGO with cysteine forms a reversible hemithioacetal (CEC, carboxyethyl cysteine) and a stable mercaptomethylimidazole adduct (MICA).

It is now well established that amino acid residues containing thiol, imidazole, and amino groups are the primary targets for RCS [82]. RCS can covalently interact with the free amino groups in nucleotide moieties, leading to DNA damage [83]. Protein and adduct nucleic acid formation is a result of the dehydration of the reversible Schiff base formed in between the amino group in a protein and the aldehyde group of RCS or the Michael addition of a nucleophilic amino acid group to the α,β-double bond of RCS, such as 4-hydroxy-2-nonenal. This forms a Michael adduct, which can then undergo cyclization to yield its hemiacetal form [84]. Thus, MGO not only affects the covalent bonding of proteins but also interacts with DNA and RNA [2]. The MGO reacts slowly with the α-amino group of amino acids but exhibits a fast reaction with cysteine or lysine, and a very rapid reaction with arginine [85]. When MGO spontaneously interacts with nucleotides and the amino acid residues of proteins (Figure 5), it forms various adducts, including hydroimidazolone, argpyrimidine, tetrahydropyrimidine, N-(1-carboxyethyl) lysine, and lysine dimers derived from MGO [86]. The accumulation of insoluble and long-lasting AGEs plays a significant role in the aging process and its progression [87]. AGEs are recognized as biomarkers linked to the onset and progression of various pathologies, including neurodegenerative diseases and cancer [2,88]. MGO-derived AGEs are implicated in the pathogenesis of various diseases impacting the brain, heart, musculoskeletal system, liver, pancreas, kidneys, and immune system [2].

AGEs can stimulate NADPH oxidase and the unregulated production of superoxide radicals (O_2_**^•−^**) in mitochondria [89,90]. Oxidative stress can also be induced by the inhibition of antioxidant enzymes caused by the reaction with α-dicarbonyl compounds [91,92].

Not only are carbonyl and dicarbonyl compounds generated by ROS, but ROS are also produced as by-products during the formation and decomposition of, for example, MGO itself. The processes leading to MGO formation—either from aminoacetone (catalyzed by semicarbazide-sensitive amino oxidase, SSAO) or from acetol (catalyzed by galactose oxidase)—are linked to the generation of hydrogen peroxide [93,94]. The conversion of MGO to pyruvate by glyoxal oxidase also generates H_2_O_2_ [95]. Nakayama et al. [96] showed that MGO reacts with H_2_O_2_ to form three types of radicals: methyl, carbon-centered, and hydroxyl radicals. The inhibition of catalase activity by carbonyl compounds, which catalyzes the conversion of H_2_O_2_ into H_2_O and O_2_ [97], points to the connection between oxidative and carbonyl stress. The auto-oxidation of aminoacetone to MGO, particularly in the presence of metal ions like iron and copper, is considered a source of carbon and O_2_**^•−^** [98,99]. Similarly, the non-enzymatic reaction between acetoacetate and MGO can lead to the formation of ROS in the presence of various proteins, including myoglobin, hemoglobin, manganese, cytochrome c, or peroxidase [100,101]. Lankin et al. [76] demonstrated that the reaction of methylglyoxal carbonyl groups with two L-lysine molecules leads to the formation of a Schiff base, dialkylimine. Subsequently, the interaction of dialkylimine with another molecule of α-oxoaldehyde results in the formation of a Schiff base cation radical and the methylglyoxal radical (MGO**^•−^**). In aqueous media, O_2_**^•−^** can reduce certain organic radicals [102]. It can be proposed that O_2_**^•−^**, acting as electron donors, interact with the MGO**^•−^**, reducing it to non-radical products. However, the enzymatic dismutation of O_2_**^•−^** by superoxide dismutase (SOD) protects the MGO**^•−^** under aerobic conditions [76]. It is probable that the oxidative modification of proteins and other biomolecules results from the local generation of O_2_**^•−^**, which affects the interaction between L-lysine residues (and potentially those of other amino acids) and α-oxoaldehydes. This approach to non-enzymatic O_2_**^•−^** generation could represent a mechanism of autocatalytic amplification in the pathophysiological effects of carbonyl stress [76].

RCS and the end products of lipoxidation (ALE) may arise in ROS mediation and by non-enzymatic lipid peroxidation [84]. Lipid peroxidation mediated by hydroxyl (HO**^•^**) or hydroperoxyl (HO_2_**^•^**) radicals leads to the abstraction of a hydrogen atom and the formation of a carbon-centered radical (R**^•^**) [103]. This carbon-centered radical then readily interacts with O_2_ and undergoes rearrangement, resulting in the formation of a peroxyl radical (ROO**^•^**). The simplest peroxyl radical is the protonated form of O_2_**^•−^**, the hydroperoxyl radical (HOO**^•^**). Although peroxyl radicals can be transformed into non-radical products, they are far more stable than either the carbon-centered radical or the related alkoxyl radicals (RO**^•^**). The H abstraction by ROO**^•^** from another lipid molecule causes the formation of hydroperoxides, ROOH, which are key intermediates of lipid peroxidation, leading to the further generation of multiple peroxidation products, among which are carbon- and oxygen-centered radicals [104]. Among the reactions leading to reactive carbonyl species formation are the β-scission of the alkoxyl radical, Hock rearrangements, and peroxyl radical cyclization [84]. The RCS formed are non-substituted α,β-unsaturated aldehydes (acrolein, crotonaldehyde, 2-nonenal), dialdehydes (glyoxal, malondialdehyde), α,β-unsaturated 4-hydroxy-alkenals (4-hydroxynonenal, 4-hydroxy-2-hexenal), and oxo-alkenals (MGO, 4-oxo-2-nonenal, and 4-oxo-2-hexenal) [105,106].

### 3.1. Hormetic Response to Subtoxic Doses of MGO and Other Carbonyl Compounds

Hormesis is defined as a process in which exposure to a low dose of a chemical agent or environmental factor, which is harmful at higher doses, induces an adaptive beneficial effect on a cell or organism [107]. In this context, small amounts of ROS can trigger an adaptive response that enhances the body’s defense mechanisms, delaying the onset of aging-related diseases and potentially extending the overall lifespan [108]. Recent studies have provided compelling evidence that reactive carbonyl compounds also exhibit hormetic effects, supporting the concept of ‘glycohormesis’. Zemva et al. [109] demonstrated in yeast that low levels of MGO activate a multi-phase adaptive response. This feedback mechanism includes a transcriptional response to prevent the excessive production of carbonyl compounds, detoxification of reactive metabolites (e.g., induction of Nrf2 and upregulation of GLO1), and damage repair through the protein quality control system (e.g., induction of molecular chaperones like HSP70 and BTN2).

Given the interconnection between oxidative and carbonyl stress, the adaptive response pathway can be outlined according to [110]. The rise in ROS levels triggers early GSH oxidation and the formation of oxidized glutathione (GSSG; resulting in a decreased GSH/GSSG ratio), followed by the activation of protein S-glutathionylation and an increase in the MGO levels. The S-glutathionylation reaction is a key response, reflecting fluctuations in the redox state of the glutathione pool [111,112]. S-glutathionylation reversibly inhibits key glycolytic enzymes, including glyceraldehyde-3-phosphate dehydrogenase (GAPDH), aldolase, phosphoglycerate kinase, pyruvate kinase, triose phosphate isomerase, and L-lactate dehydrogenase [113,114]. Reduced GAPDH activity is strongly correlated with MGO formation [115]. When sufficient GSH is available, the increased formation of the MGO-GSH hemithioacetal and, subsequently, S-lactoylglutathione by GLO1 occurs. GLO2 has the ability to catalyze S-glutathionylation using S-lactoylglutathione as a substrate [116,117], slowing down glucose catabolism. Thus, the formation of MGO will also promote the pentose phosphate pathway, the primary source of NADPH required for the reduction of oxidized glutathione (GSSG). An increase in the GSH/GSSG ratio enhances both the ability of GSH to directly combat ROS and RCS, and the activation of the deglutathionylase activity of glutaredoxins (GRXs), which helps restore normal metabolism [118].

Based on the above, disruption of the mechanisms responsible for RCS detoxification, including MGO, would impair the adaptive response to stress. GAPDH also possesses RNA-regulatory activity, since evidence suggests that it acts as a regulatory RNA-binding protein when its enzymatic activity is inhibited by MGO in neural precursor cells [119], similar to the findings in T cells [120]. The regulation of GLO1 expression is negatively affected by HIF1-α under hypoxic conditions [121]. As a result, hypoxia may induce dicarbonyl stress by accelerating glycolysis, which simultaneously increases MGO production while decreasing GLO1 activity [60]. Elevated MGO concentrations—whether due to increased production or decreased GLO1 activity associated with aging—can gradually lead to the accumulation of AGEs in various tissues, including the brain [122,123]. Furthermore, evidence suggests that the GLO1 gene is a key hotspot for copy-number variations linked to multidrug resistance in cancer chemotherapy [124]. GLO1 is therefore a key enzyme in MGO detoxification, while GLO2 plays a crucial role in maintaining the cell’s redox balance.

### 3.2. Agents That Mitigate MGO Accumulation

The dysfunction of antioxidant enzymes caused by elevated MGO levels plays a key role in the pathogenesis of several chronic diseases [94,125]. Therapies designed to restore the function of these enzymes and reduce MGO levels may effectively mitigate oxidative stress and its harmful effects on the body.

Similarly, glyoxal, another highly reactive dialdehyde capable of forming AGEs, has been shown to react with SOD1, forming stable adducts [126]. Furthermore, ex vivo experiments indicate that SOD1 extracted from the erythrocytes of diabetic patients is significantly more glycated and exhibits lower enzymatic activity compared to controls [127]. The glutathione peroxidase and glutathione conjugation system plays a crucial role in reducing oxidative stress, particularly lipid peroxidation, both of which are closely linked to the formation of RCS. The system includes glutathione peroxidase (GPx) and glutathione reductase (GR). In the decomposition of H_2_O_2_ or other organic peroxides (ROOH), two molecules of GSH reduce the substrate to H_2_O or the corresponding non-radical alcohol (ROH) while regenerating the enzyme and forming GSSG as a by-product [19]. It is GPx4 that has the unique ability to reduce complex lipid hydroperoxides [128], which is beneficial, unless the enzyme is inactivated by elevated concentrations of peroxides or RCS itself [129]. GR inactivation can also occur through an NADPH-independent mechanism [130]. Both MGO and all the RCSs conjugate with the thiol group of GSH via Michael addition, forming either hemithioacetal adducts or RCS-modified glutathione adducts, depending on the reaction rate [131]. The conjugation of RCSs can also be catalyzed by cytosolic glutathione-S-transferase in a phase II reaction [132] (GST; EC 2.5.1.18). When the pentose phosphate pathway is sufficiently strengthened, it enables the efficient catalysis of GSH conjugation reactions, especially for metabolites formed by cytochrome P450 [133].

Antioxidants such as sulforaphane and resveratrol have the ability to inhibit advanced glycation end product (AGE)-mediated reactions. Studies have demonstrated that sulforaphane protects against the cytotoxic effects of MGO in neuroblastic and cardiomyocyte cells by upregulating the glyoxalase system and preventing the activation of stress pathways [134,135]. Resveratrol (3,5,4′-trihydroxy-trans-stilbene), a polyphenolic compound found in berries, red wine, and peanuts, is a well-known antioxidant that has been shown to scavenge MGO and improve mitochondrial function. Resveratrol also protects β-cells, mouse oocytes, and blastocysts during embryonic development from MGO-induced glycation [136,137,138,139]. It reduces high blood glucose levels, excess lipids, fatty liver, and complications associated with diabetes mellitus [140]. Other substances, such as limonene, honokiol [141], Nrf2, carnosic acid, 1-(2-cyano-3,12,28-trioxolan-1,9(11)-died-28-yl)-1H-imidazole [142], and fisetin [143], help attenuate MGO accumulation by enhancing its detoxification. Additionally, DNA damage-inducible transcript 3 deficiency [144,145] and the role of cytochrome c peroxidase in the enzymatic activity of D-erythroascorbate peroxidase contribute to the regulation of physiological concentrations of MGO [146].

## 4. The Role of MGO in Cell Signaling and Apoptosis

Increased MGO formation can have detrimental effects, particularly in organs and tissues with high demands for glucose uptake and utilization as an energy source. Moreover, the link between glycation and oxidative stress, along with the promotion of additional RCS formation due to oxidative stress, contributes to the disruption of cellular homeostasis and may lead to pathological conditions and diseases. The effects of MGO are mediated through multiple signaling pathways.

### 4.1. PERK-eIF2α Signaling Pathway

The PERK-eIF2α pathway plays a crucial role in the cellular response to stress in the endoplasmic reticulum (ER), a process known as the unfolded protein response (UPR) [147,148]. This pathway is activated when misfolded proteins accumulate in cells and serves as a primary mechanism for restoring cellular homeostasis [149,150,151]. It is an essential component of protein homeostasis, ensuring the refolding or removal of misfolded proteins while simultaneously triggering low-level inflammation [152]. PERK (protein kinase RNA-like endoplasmic reticulum kinase) is a key sensor of ER stress, activated by protein misfolding, which can also be induced by methylglyoxal (MGO) [153,154,155]. Through oxidative stress and protein glycation, MGO disrupts normal protein folding, triggering PERK activation [153,156,157].

In turn, PERK phosphorylates eukaryotic initiation factor 2α (eIF2α), leading to a reduction in protein translation, thereby alleviating cellular stress [158,159]. However, PERK also promotes the translation of specific mRNAs, such as ATF4, which regulates genes involved in antioxidant responses, protein folding, and apoptosis. In the case of prolonged stress induced by MGO, the activation of ATF4 can lead to the expression of genes that promote apoptosis [160,161]. MGO increases the production of reactive oxygen species (ROS), contributing to oxidative damage and sustained ER stress [162,163]. This chronic stress ultimately activates pro-apoptotic factors, such as C/EBP homologous protein, which can trigger cell death [164]. Protein misfolding is an initiating event in various neurodegenerative disorders, also referred to as protein misfolding disorders (PMDs) [94,165].

### 4.2. IRE1-JNK Signaling Pathway

IRE1 (inositol-requiring enzyme 1) is one of the three major sensors of ER stress [166,167]. During ER stress, such as that induced by methylglyoxal, IRE1 is activated through dimerization and autophosphorylation [154]. Methylglyoxal typically induces this stress by modifying proteins and disrupting their folding, thereby activating IRE1 in the UPR process [168]. Upon activation, IRE1 splices the mRNA of X-box binding protein 1 (XBP1), leading to the formation of the transcription factor sXBP1 [169,170,171]. This factor enhances the expression of genes involved in protein folding, ER-associated degradation, and lipid synthesis, representing an adaptive cellular response to ER stress [172,173]. Additionally, IRE1 activates Jun N-terminal kinase (JNK) through the interaction of its cytoplasmic domain with adaptors such as TRAF2 (TNF receptor-associated factor 2) [174,175]. JNK, a member of the mitogen-activated protein kinase (MAPK) family, regulates cellular responses to stress, including inflammation, oxidative stress, and apoptosis [176,177]. Methylglyoxal-induced oxidative stress exacerbates ER stress and activates JNK via IRE1 [178,179]. The prolonged activation of JNK can promote apoptosis by phosphorylating pro-apoptotic proteins like Bim and inhibiting anti-apoptotic proteins such as Bcl-2, ultimately leading to programmed cell death [180]. The axis between IRE1 and JNK is especially critical in situations where the ER stress remains unresolved, causing the cell to shift from an adaptive response to an apoptotic one [181,182]. Oxidative stress further amplifies JNK-mediated inflammation, making this pathway pivotal in diseases linked to chronic inflammation and apoptosis, such as diabetes, neurodegenerative diseases, and cardiovascular disorders [183].

### 4.3. RAGE Signaling Pathway

RAGE (receptor for advanced glycation end products) is a member of the immunoglobulin superfamily, expressed on the surface of various cell types, including muscle cells, vascular wall cells, monocytes, macrophages, and brain cells such as astrocytes, oligodendrocytes, and neurons [88,184]. RAGE plays a key role in the cell’s response to methylglyoxal and other reactive dicarbonyl compounds [185,186]. The activation of RAGE occurs when proteins and lipids are modified by MGO, leading to the formation of AGEs, which then bind to this receptor [187,188]. Upon interaction, RAGE undergoes a conformational change, triggering the activation of various signaling cascades [189], which lead to inflammatory responses, oxidative stress, and tissue damage [190]. The primary signaling pathways activated by RAGE include mitogen-activated protein kinases (MAPKs) such as ERK1/2, p38, and JNK, as well as nuclear factor kappa B (NF-κB) [191,192,193]. The activation of NF-κB enhances the expression of pro-inflammatory cytokines, such as TNF-α, IL-1β, and IL-6, along with adhesion molecules, thereby amplifying inflammatory processes [194]. NF-κB also regulates the expression of RAGE itself, suggesting a positive inflammatory feedback loop [195]. Additionally, RAGE enhances ROS production through the activation of NADPH oxidase [88,196]. These effects may contribute to impaired neuronal plasticity, a characteristic feature of many psychiatric and cognitive disorders, as well as pain and nociception [168,188,197]. They may also trigger apoptotic mechanisms [198]. Furthermore, RAGE activates several pathways, including protein kinase C, p21, extracellular-regulated kinase (ERK) 1/2, p38, and JNK, all of which are linked to the translocation of NF-κB to the nucleus. NF-κB, in turn, induces the expression of various inflammatory cytokines (e.g., IL-6, IL-1β, TNF-α), adhesion molecules, vascular endothelial growth factor (VEGF), and even RAGE itself [196,199]. RAGE downregulates GLO1 expression, and RAGE knockout prevents MGO formation [200].

This process is critical in several pathophysiological conditions. In diabetes mellitus, excessive MGO production stimulates AGE formation and RAGE activation, contributing to complications such as diabetic neuropathy, nephropathy, and retinopathy [201,202,203]. Similarly, in cardiovascular diseases, RAGE activation promotes endothelial dysfunction, vascular inflammation, and atherogenesis [204]. In neurodegenerative diseases, such as Alzheimer’s disease, RAGE mediates neuroinflammation, leading to progressive cognitive decline and neurodegeneration [205]. The inhibition of RAGE or its downstream signaling pathways offers a promising therapeutic approach for MGO- and AGE-related diseases [206,207]. These therapeutic strategies may include RAGE antagonists, ROS production inhibitors, or anti-inflammatory agents that could mitigate the pathological effects caused by activation of this signaling pathway [208,209]. RAGE antagonists have even been shown to reverse memory impairment induced by MGO [88].

### 4.4. JAK/STAT Signaling Pathway

The JAK/STAT pathway plays a crucial role in inflammatory processes and apoptosis [210,211]. Activation of this pathway is triggered by oxidative stress induced by MGO [110]. The subsequent interaction of AGE with the RAGE receptor initiates signaling that activates the JAK/STAT cascade [198,212]. Following phosphorylation by Janus kinase (JAK), STAT proteins (particularly STAT1, STAT3, and STAT5) dimerize and translocate to the nucleus, where they regulate the expression of genes involved in inflammation and apoptosis [213]. In this context, MGO stimulates the production of pro-inflammatory cytokines, such as IL-6, TNF-α, and interferons, contributing to chronic inflammation. This pathway plays a significant role in conditions like diabetes, cardiovascular diseases, and cancer [212,214]. MGO-induced oxidative stress can also enhance the expression of pro-apoptotic genes through the JAK/STAT pathway, thereby accelerating cell death in tissues sensitive to oxidative damage, such as endothelial cells, neurons, and pancreatic β-cells [215,216]. Dysregulation of this pathway has significant implications in MGO-associated diseases, including diabetes, where JAK/STAT activation contributes to insulin resistance; cardiovascular disease, where it promotes vascular inflammation and atherosclerosis; and cancer, where it may facilitate the tumor microenvironment and tumor progression [217,218,219]. From a therapeutic standpoint, targeting the JAK/STAT pathway with Janus kinase inhibitors, already used in the treatment of rheumatoid arthritis and certain cancers, offers a promising strategy to reduce the inflammatory and apoptotic effects of MGO in various diseases [220,221,222].

### 4.5. NF-κB Signaling Pathway

Exposure to MGO results in the phosphorylation and degradation of inhibitors of NF-κB (IκB), leading to the release of NF-κB dimers (p65/p50), which then translocate to the nucleus and activate the transcription of pro-inflammatory genes [223]. The NF-κB pathway promotes inflammatory complications in conditions such as neuropathy, nephropathy, and retinopathy [203,224], and it contributes to endothelial dysfunction and atherosclerosis [225]. In neurodegenerative diseases like Alzheimer’s disease, activation of the NF-κB pathway triggers neuroinflammation and neuronal damage, exacerbating cognitive decline [226,227]. Activation of the NF-κB pathway leads to the increased production of ROS, creating a positive feedback loop that amplifies oxidative stress and promotes cell apoptosis, contributing to tissue damage and organ failure [228]. From a therapeutic standpoint, inhibiting the NF-κB pathway could offer benefits in diseases associated with excessive MGO exposure [229,230]. NF-κB inhibitors or compounds that reduce oxidative stress, such as curcumin, may help mitigate the inflammatory and oxidative damage caused by MGO [231].

The NF-κB and Nrf2 (nuclear factor erythroid 2-related factor 2) pathways influence one another [232] to form a coordinated response to inflammation or oxidative stress. Recently, Wei et al. [233] showed that MGO may hold therapeutic potential for treating autoimmune CNS diseases, such as multiple sclerosis, primarily by activating the Nrf2 pathway. MGO was shown to suppress microglial activation and reduce autoimmune processes in the brain, particularly in the experimental autoimmune encephalomyelitis (EAE) model, which mimics multiple sclerosis. It was found to activate the Nrf2-IκBζ pathway, resulting in decreased production of pro-inflammatory cytokines and alterations in microglial cell polarization—specifically reducing M1 polarization, which is linked to inflammation.

### 4.6. Intrinsic and Extrinsic Pathways of Apoptosis

Under excessive stress, such as prolonged hypoxia, nutrient deprivation, or increased ROS accumulation, cell death signals may be triggered instead of adaptive responses [234]. The intrinsic apoptosis pathway is activated by internal signals resulting from cellular stress, DNA damage, or mitochondrial dysfunction [235]. Mitochondria play a central role in this pathway, responding to damage by releasing molecules like cytochrome c into the cytosol [236,237]. This process is regulated by the balance between pro-apoptotic (Bax, Bak) and anti-apoptotic proteins (Bcl-2, Bcl-xL) within the Bcl-2 family [238]. When pro-apoptotic proteins dominate, the outer mitochondrial membrane becomes permeabilized, leading to the release of cytochrome c [239], which then binds to Apaf-1 (apoptotic protease-activating factor-1) in the cytoplasm, forming the apoptosome [240,241]. This complex activates caspase-9, which in turn activates effector caspases, such as caspase-3 and -7, which are responsible for degrading cellular structures and triggering cell death [242,243].

The extrinsic pathway of apoptosis is activated by external signals originating from other cells or the surrounding environment [244,245,246]. This pathway is initiated when ligands bind to specific receptors on the cell surface, such as the death receptor (Fas) or the tumor necrosis factor (TNF) receptor [247,248]. This binding triggers a signaling cascade that activates caspase-8, which then activates effector caspases (e.g., caspase-3), which degrade cellular components and ultimately lead to cell death [249,250,251]. MGO can also indirectly influence the extrinsic apoptosis pathway by promoting the generation of ROS and oxidative stress, which in turn amplify the apoptotic response [252]. Additionally, glycation reactions induced by MGO can alter the structure of death receptors or their associated signaling molecules, potentially increasing or decreasing the sensitivity of cells to extrinsic apoptotic signals [253,254,255].

## 5. MGO and Its Role in Disease Pathology

**Figure 6:**
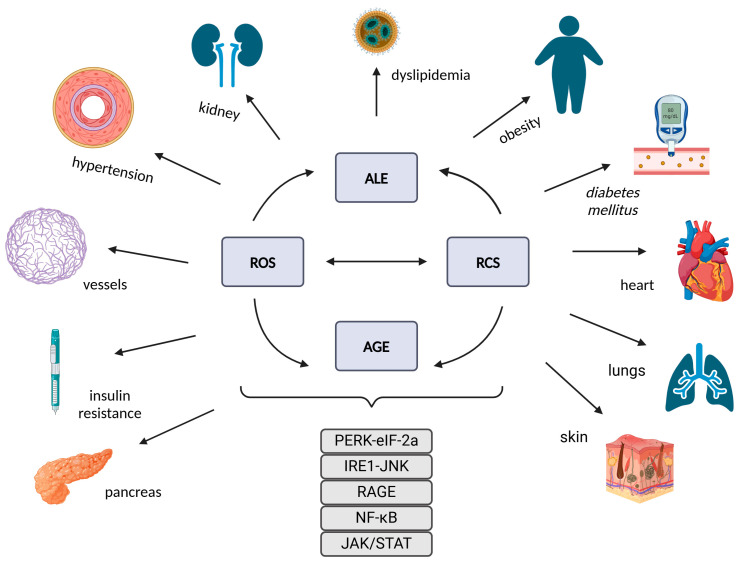
The impact on the tissues, organs, and systems through the activation of signaling pathways and the formation of advanced lipoxidation and glycation end products (ALEs and AGEs). This occurs due to the mutual potentiation of oxidative and carbonyl stress, resulting in the generation of reactive oxygen species (ROS) and reactive carbonyl species (RCS).

Methylglyoxal (MGO) disrupts the function of multiple organ systems, including the heart, blood vessels, brain, kidneys, liver, lungs, and skin [2,256,257]. Despite varying causative factors and clinical manifestations, these diseases share common pathogenic features (Figure 6), including mitochondrial dysfunction, endoplasmic reticulum stress, ROS accumulation, protein homeostasis disorders, and neuroinflammation [88,258]. Therefore, understanding the mechanisms and sites of MGO’s action is a critical research focus for the prevention and treatment of various systemic diseases.

### 5.1. Pancreas

MGO contributes to oxidative stress in pancreatic β-cells by reacting with lipids, proteins, and DNA, causing damage and dysfunction [259,260]. It triggers inflammatory responses in the pancreas through the activation of pro-inflammatory cytokines such as TNF-α, IL-1β, and IL-6 [214,261]. This inflammation, along with oxidative stress, can impair β-cell function and contribute to the development of diabetic complications, including the induction of the intrinsic apoptosis pathway [262,263].

### 5.2. Liver

Activation of the NF-κB and JNK pathways is associated with hepatocyte apoptosis [194,264]. Oxidative stress and apoptosis induced by MGO play a significant role in the development and progression of liver diseases [201,265,266]. In nonalcoholic steatohepatitis (NASH), MGO exacerbates insulin resistance and metabolic disorders, leading to increased AGE formation and disease progression [168]. In liver cirrhosis, MGO contributes to liver tissue damage and fibrotic tissue formation [201].

### 5.3. Kidney

In diabetic nephropathy, MGO contributes to the formation of AGEs, which exacerbate oxidative stress and inflammation, leading to damage to the renal glomeruli and tubules [267,268]. AGEs increase capillary permeability, resulting in proteinuria (the presence of protein in the urine) [269]. They also promote inflammation and the formation of fibrotic tissue in the kidneys, accelerating the progression of diabetic nephropathy [170,179]. In chronic kidney disease (CKD), MGO plays a role in the progressive decline of kidney function through various mechanisms, including inflammation and fibrosis [270,271,272].

### 5.4. Lungs

Increased oxidative stress due to MGO leads to alveolar tissue destruction, and inflammatory responses exacerbate airway narrowing, contributing to progressive obstruction and impaired lung ventilation, which are key features of chronic obstructive pulmonary disease (COPD) [273]. In asthma, MGO plays a significant role in the development of airway inflammation, which leads to bronchial hyperresponsiveness and narrowing [274,275]. The oxidative stress caused by MGO can worsen asthma symptoms and increase the severity of attacks [276,277]. Inflammatory responses further promote airway remodeling, causing narrowing and decreased elasticity, thus impairing lung function and the ability to breathe effectively [278].

### 5.5. Skin

In the skin, AGEs negatively impact the integrity of collagen and elastin, which are essential for maintaining the firmness and elasticity of the dermis. This contributes to the formation of fine lines and wrinkles, characteristic of skin aging [279,280]. MGO-induced AGE formation also increases oxidative stress, triggering inflammatory responses and damaging skin cells, thereby accelerating the skin’s aging process and deteriorating its appearance [186,281].

### 5.6. Heart

MGO induces cardiomyocyte apoptosis by activating pro-apoptotic proteins such as Bax while inhibiting anti-apoptotic factors like Bcl-2 [282]. This mechanism leads to a reduction in the cardiomyocyte number, impairing cardiac function [283]. Additionally, MGO activates inflammatory pathways, including NF-κB, which increases the production of pro-inflammatory cytokines such as TNF-α and IL-6. This further damages cardiomyocytes and promotes the progression of cardiovascular disease [233,284]. In cardiomyopathies, MGO contributes to disease development through apoptosis and inflammation [285]. MGO-induced apoptosis leads to the loss of functional cardiomyocytes, weakening the heart muscle and impairing its ability to pump blood effectively [286]. Inflammation caused by MGO further disrupts cardiac tissue remodeling, contributing to hypertrophy and fibrotic changes that reduce cardiac performance [287,288]. In ischemic heart disease (IHD), oxidative stress and inflammation damage coronary vessels and cardiomyocytes [201,289,290]. AGEs generated by MGO cause vessel stiffness and narrowing, impairing the blood flow to cardiac tissue and leading to ischemic damage [291]. Additionally, MGO-induced inflammatory processes may contribute to the development of atherosclerosis, which increases the risk of myocardial infarction [292].

### 5.7. Vessels

Endothelial dysfunction is a key mechanism through which MGO affects blood vessels [267]. MGO reduces the availability of nitric oxide (NO), impairing vasodilation and blood pressure regulation [293]. This reduction in NO disrupts the balance between vasodilation and vasoconstriction, leading to increased vascular resistance and contributing to hypertension [294,295]. Additionally, MGO directly damages endothelial cells, promoting endothelial dysfunction and initiating inflammatory processes [296,297]. MGO activates inflammatory pathways, such as NF-κB, resulting in increased production of pro-inflammatory cytokines like TNF-α and IL-6 [298]. Chronic inflammation within the vascular wall, along with lipid accumulation and monocyte migration, heightens the risk of atherosclerotic plaque formation and the development of atherosclerosis [230,299]. Furthermore, MGO-induced apoptosis contributes to the weakening of the vascular wall, exacerbating the progression of vascular disease [300].

### 5.8. Metabolic Syndrome

MGO plays a central role in the development of metabolic syndrome, a cluster of risk factors, including obesity, insulin resistance, hypertension, dyslipidemia, and diabetes mellitus [267,301]. While the pathophysiology of metabolic syndrome is multifactorial, numerous studies indicate that insulin resistance—induced by increased fatty acid metabolism and oxidative stress—activates the AGE, PKC, hexosamine, and NF-κB pathways. The glycation of insulin by MGO contributes to insulin resistance by impairing glucose uptake and reducing insulin clearance. MGO directly modifies insulin molecules, disrupting their function and signaling pathways [302]. Additionally, MGO interacts with β-cells and endothelial cells, leading to swelling and apoptosis, which further impair cellular function [303].

In pancreatic β-cells, MGO leads to reduced insulin secretion [304]. The plasma MGO levels in patients with type 1 diabetes mellitus are five to six times higher compared to healthy individuals, while in type 2 diabetes mellitus, these levels are typically two to three times higher [4]. The pathophysiology of diabetes mellitus resembles a state of chronic inflammation, with elevated levels of inflammatory cytokines such as interleukin-6 (IL-6) and tumor necrosis factor-α (TNF-α) in patients with type 2 diabetes. The increased expression of IL-1β and TNF-α has been linked to the development of microvascular damage and complications resembling those of diabetes mellitus in animal models [305]. Diabetic patients with acute coronary syndrome (ACS) show higher CRP levels than non-diabetic ACS patients do, suggesting that the inflammation in diabetics with ACS is more severe, which increases the risk of atherosclerotic plaque rupture [306]. Carbonyl stress, a key pathogenic mechanism, is associated with diabetic complications. Elevated carbonyl stress levels in patients with type 2 diabetes mellitus may increase their susceptibility to complications such as ACS, hypertension, neuropathy, and nephropathy [303,307].

Hyperglycemia and dyslipidemia are interrelated causes and consequences of type 2 diabetes mellitus. The resulting metabolic changes affect lipid metabolism, leading to increased triglyceride levels and decreased HDL cholesterol levels, which in turn heighten the risk of atherosclerosis [308].

### 5.9. Brain and Nervous Tissue

Given the high energy expenditure and the fact that aerobic glucose metabolism is a key energy source, glycolysis may contribute significantly to MGO formation in the brain [309]. Oxidative stress disrupts mitochondrial function, decreasing the energetic efficiency of neurons and increasing their vulnerability to damage [310,311,312]. In mice, the plasma MGO levels range between 0.1 and 0.5 μM, while the cerebrospinal fluid levels can reach up to 5 μM [313,314]. MGO is linked to microvascular dysfunction, and maintaining the integrity of the blood–brain barrier (BBB) is essential for optimal brain function [267]. Elevated MGO concentrations impact the blood–brain barrier (BBB) and contribute to altered brain function, which manifests as a more rapid memory decline [315], anxiety-like behaviors [316], and the progression of neurodegenerative diseases [317,318]. Clinical studies have revealed that patients with neurodegenerative diseases exhibit increased MGO levels in their cerebrospinal fluid [17]. Moreover, older individuals with higher serum MGO concentrations have shown faster cognitive decline [319].

MGO has the potential to disrupt the proper functioning of astrocytes [320] and activate inflammatory pathways through the stimulation of microglia [321,322]. Once activated, microglia release pro-inflammatory cytokines such as TNF-α and IL-6, which intensify brain inflammation and cause further neuronal damage. This persistent inflammation plays a key role in the progression of neurodegenerative diseases [323].

MGO is also known to influence neurotransmission by altering glutamatergic [320] and GABAergic synapses [316]. Several studies have linked the MGO levels, GLO1 activity, and their role in the pathogenesis of various conditions, including schizophrenia, autism, anxiety, depression, sleep disorders, and pain phenotypes [316,324,325,326,327]. The binding of MGO to receptors such as GABAA, Nav1.8, TRPV, and TRPA1 is crucial for pain perception. Specifically, the binding to Nav1.8, a voltage-gated sodium channel, enhances nociceptive neuronal conduction and contributes to hyperalgesia in diabetic neuropathy [326]. MGO may also affect the transient receptor potential ankyrin receptor 1 (TRPA1), which, when activated, induces pain [328].

Brain inflammation is also recognized as a contributing mechanism in epilepsy [329]. Given these findings, the AGE/RAGE pathway may serve as a detrimental mechanism for signal transduction during epileptic seizures [330]. Changes in GLO1 expression and activity can influence susceptibility to epilepsy by modulating the concentration of endogenous MGO in the brain [331]. Elevated serum MGO levels are also observed in older individuals and are linked to increased cognitive decline [325]. Studies in older adults without dementia have shown a positive correlation between the serum MGO concentration and the rate of cognitive decline [319,330].

In Alzheimer’s disease, MGO promotes the formation of beta-amyloid plaques and tau protein tangles, which are hallmarks of the disease [332,333]. The AGEs generated by MGO bind to beta-amyloid, accelerating its aggregation and deposition in the brain [17,334,335]. Additionally, MGO enhances inflammation and oxidative stress, impairing cognitive function and accelerating disease progression [314,336]. In individuals with Alzheimer’s disease, the levels of MGO in the cerebrospinal fluid can be up to twice as high as those in healthy individuals of the same age, reaching concentrations as high as 20 μM [337]. In the early stages of Alzheimer’s disease, the GLO1 activity is increased, but it gradually decreases in the middle and late stages [338]. Inadequate glyoxalase system function in Alzheimer’s patients also raises the GSSG/GSH ratio, potentially exacerbating the severity of the disease [125].

In Parkinson’s disease, MGO contributes to the loss of dopaminergic neurons in the substantia nigra, a key mechanism behind the motor symptoms of the disease [339,340]. Oxidative stress and inflammation induced by MGO accelerate the degeneration of these neurons, impairing motor control and leading to typical Parkinson’s disease symptoms such as tremor, rigidity, and bradykinesia [339,341,342]. This degeneration may be linked to reduced GLO1 activity, which has also been observed in some patients with Parkinson’s disease (PD), schizophrenia, and amyotrophic lateral sclerosis (ALS) [343].

### 5.10. Cancer

Studies from the 1990s suggested that MGO can suppress tumor cell growth by inhibiting glycolysis and mitochondrial respiration specifically in malignant cells [344]. This beneficial effect was further confirmed through the intraperitoneal injection of MGO in a mouse model of sarcoma. The expression and activity of GLO1 in certain cancer cells have been shown to correlate with tumor progression, indicating a significant role for GLO1 in carcinogenesis [319,345]. While GLO1 was initially considered an oncogene due to its increased expression and amplification in cancer, genetic studies have instead identified GLO1 as a tumor suppressor gene [346].

## 6. Conclusions

MGO is a reactive dicarbonyl compound formed as a by-product of glucose metabolism and other metabolic pathways. It plays a significant role in the pathogenesis of various chronic diseases, including diabetes mellitus, neurodegenerative disorders such as Alzheimer’s and Parkinson’s diseases, and cardiovascular diseases. As a potent glycotoxin, MGO causes protein glycation, leading to the formation of advanced glycation end products (AGEs). The oxidative and carbonyl stress induced by MGO are mediated through multiple signaling pathways, including NF-κB, MAPK, and Nrf2. NF-κB activation triggers the transcription of pro-inflammatory cytokines and adhesion molecules, thereby amplifying the inflammatory response. On the other hand, the MAPK pathways play a crucial role in regulating cell proliferation and apoptosis. Nrf2, a key regulator of antioxidant genes, is activated in response to oxidative stress. However, elevated levels of MGO can disrupt this pathway, leading to reduced production of antioxidant enzymes. The interplay between MGO, antioxidant enzymes, and these signaling pathways forms a complex biochemical framework that is vital for understanding the mechanisms behind the development of chronic diseases. Furthermore, the link between oxidative and carbonyl stress highlights connections that have yet to be fully explored in many diseases. This, however, opens up opportunities for monitoring the MGO levels and related metabolic pathway markers in other diseases. Additionally, it paves the way for identifying potential biomarkers—similar to antioxidant enzymes like SOD—that could be crucial for disease prognosis or evaluating the effectiveness of treatments. The interactions among these factors offer valuable insights for the development of new therapeutic strategies aimed at improving the clinical outcomes in patients affected by MGO-associated diseases.
